# Retinoic acid decreases ATF-2 phosphorylation and sensitizes melanoma cells to taxol-mediated growth inhibition

**DOI:** 10.1186/1750-2187-3-3

**Published:** 2008-02-12

**Authors:** Ying Huang, Jennifer Minigh, Sarah Miles, Richard M Niles

**Affiliations:** 1 Department of Biochemistry and Microbiology, Joan C. Edwards School of Medicine, Marshall University, One John Marshall Drive – BBSC, Huntington, WV, 25755, USA

## Abstract

Cutaneous melanoma is often resistant to chemo- and radiotherapy. This resistance has recently been demonstrated to be due, at least in part, to high activating transcription factor 2 (ATF-2) activity in these tumors. In concordance with these reports, we found that B16 mouse melanoma cells had higher levels of ATF-2 than immortalized, but non-malignant mouse melanocytes. In addition, the melanoma cells had a much higher amount of phosphorylated (active) ATF-2 than the immortalized melanocytes. In the course of determining how retinoic acid (RA) stimulates activating protein-1 (AP-1) activity in B16 melanoma, we discovered that this retinoid decreased the phosphorylation of ATF-2. It appears that this effect is mediated through p38 MAPK, because RA decreased p38 phosphorylation, and a selective inhibitor of p38 MAPK (SB203580) also inhibited the phosphorylation of ATF-2. Since ATF-2 activity appears to be involved in resistance of melanoma to chemotherapy, we tested the hypothesis that treatment of the melanoma cells with RA would sensitize them to the growth-inhibitory effect of taxol. We found that pretreatment of B16 cells with RA decreased the IC_50 _from 50 nM to 1 nM taxol. On the basis of these findings and our previous work on AP-1, we propose a model in which treatment of B16 cells with RA decreases the phosphorylation of ATF-2, which results in less dimer formation with Jun. The "freed-up" Jun can then form a heterodimer with Fos, resulting in the increased AP-1 activity observed in RA-treated B16 cells. Shifting the balance from predominantly ATF-2:Jun dimers to a higher amount of Jun:Fos dimers could lead a change in target gene expression that reduces resistance to chemotherapeutic drugs and contributes to the pathway by which RA arrests proliferation and induces differentiation.

## Background

The incidence of cutaneous melanoma has been rapidly increasing in the past few years. In its early stages, melanoma is curable in most cases by surgery; but once metastases develop, the median survival for patients is only 8.5 months. Treatment of patients with metastatic melanoma has been problematic because of its poor response to chemo- and radiotherapy. Recently, it has been found that activating transcription factor 2 (ATF-2) is responsible, at least in part, for resistance of melanoma to chemo- and radiotherapy [1]. Bhoumik et al., 2001 [2] reported that blocking ATF-2 transcriptional activity by using an ATF-2-derived peptide could sensitize melanoma cells to apoptosis induced either by chemotherapeutic drugs, or by inhibitors of stress kinases.

ATF-2 is a member of the ATF/CREB family of basic region leucine zipper (bZIP) proteins. Jun and Fos bZIP families, together with ATF-2, constitute the activating protein-1 (AP-1) transcription factor family. AP-1 transcription factors mediate gene regulation in response to specific growth factors, cytokines, tumor promoters, carcinogens, and oncoproteins. ATF-2 has been implicated in modulating melanoma proliferation [3] and resistance to chemo- and radiotherapy [1,4]. Under nonstressed conditions, ATF-2 is transcriptionally inactive because of its intramolecular inhibition, in which the ATF-2 activation domain and bZIP domain specifically bind to each other [5]. ATF-2 is known to acquire its transcriptional activity upon phosphorylation by MAP kinases, including JNK and p38 [5,6]. Phosphorylation at two threonine sites within the N-terminal activation domain leads to ATF-2 conformational changes, which releases the intramolecular inhibition.

Retinoids have been shown to inhibit proliferation and induce differentiation in a variety of cancer cell lines and mouse human tumor xenografts [7-9]. Some mouse and human melanoma cell lines are sensitive to the growth inhibitory and pro-differentiating effects of RA [10]. In B16 mouse melanoma cells, *all*-*trans*-RA inhibits both anchorage-dependent and -independent growth and stimulates melanin production [11]. Previously, our laboratory reported that RA induced a three to four-fold increase in AP-1 transcriptional activity [12]. This RA-induced AP-1 transcriptional activity plays an important role in the biological changes induced by this retinoid in B16 melanoma cells because blocking AP-1 transcriptional activity by a dominant negative c-Fos significantly decreases the sensitivity to RA-dependent cell growth arrest and differentiation [13].

In studying the molecular mechanism involved in RA-induced AP-1 transcriptional activity, we found that RA did not increase the expression of any of the Fos or Jun family members. Therefore, we investigated whether RA altered the expression of the AP-1 family member ATF-2. In this report we demonstrate that ATF-2 is expressed at a higher level in B16 melanoma cells when compared with an immortalized, but non-malignant, mouse melanocyte cell line. In addition, a much greater amount of phosphorylated ATF-2 protein (active) is found in B16 cells, compared with the non-malignant cells. RA treatment of B16 melanoma cells reduced ATF-2 phosphorylation, and evidence was obtained that this action was mediated through the inhibition of p38 MAP kinase activation. Because active ATF-2 has been implicated in melanoma resistance to chemotherapy, we determined whether RA inhibition of ATF-2 phosphorylation might sensitize B16 cells to the chemotherapeutic agent taxol. Pretreatment of B16 cells with RA significantly lowered the concentration of taxol required to achieve a 50% reduction in tumor cell growth.

## Results

### Expression and phosphorylation of ATF-2 protein in malignant mouse melanoma cells versus non-malignant mouse melanocytes

Previous work from our laboratory has shown that RA induces AP-1 transcriptional activity in B16 mouse melanoma cells [12,13]. However, RA does not increase the expression of any of the Fos or Jun family members. Therefore, we examined whether RA altered the ATF-2 member of the greater AP-1 family of transcription factors.

**Figure 1:**
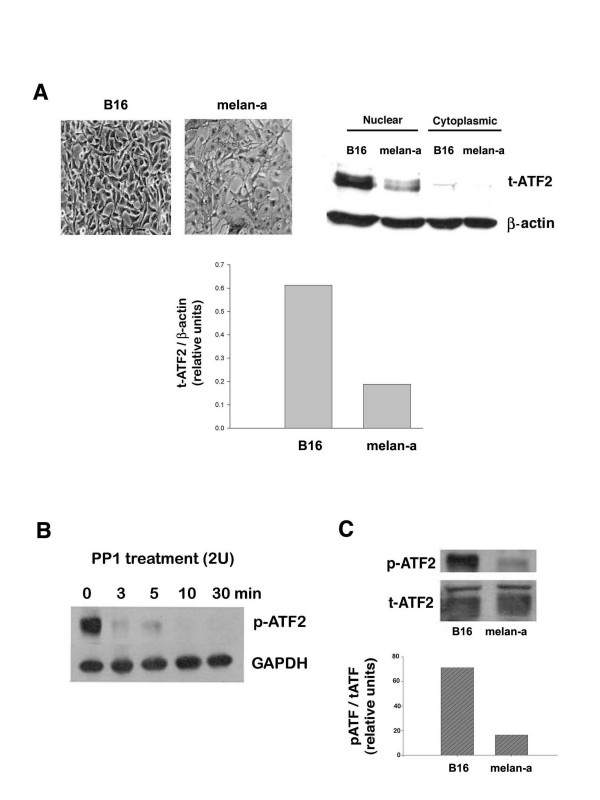
**ATF-2 expression and phosphorylation in B16 malignant melanoma vs. non-malignant melan-a mouse melanocytes**. A. Subcellular distribution and relative amount of ATF-2 in B16 mouse melanoma compared with nonmalignant melan-a cells. B16 cells and Melan-a cells were harvested at 80% confluence, and cytoplasmic and nuclear protein were isolated using Pierce NE-PER™ nuclear and cytoplasmic extraction reagents described in Materials and Methods. Cytoplasmic and nuclear proteins (10 μg) from both cell lines were analyzed by western blotting using polyclonal ATF-2 antibody. The autoradiogram (top right panel) was scanned using a Molecular Dynamics densitometer, and after correcting for the amount of β-actin, the relative amount of total ATF-2 in each sample was determined (bottom). The data shown are from a representative experiment, which was replicated three additional times with similar results. The top left panel illustrates the morphology of the two different cell lines prior to harvest (phase contrast, 20X). B. Phosphatase digestion of ATF-2. Nuclear extracts prepared from B16 cells were treated with 2 units of PP1 at 37°C. The reaction was stopped by the addition of SDS-sample buffer at the indicated incubation times. The samples were boiled, then blotted and detected using phospho-ATF-2 antibody. GAPDH was used as an internal control. The data are representative of three individual experiments with similar results. C. Relative amount of phospho- ATF-2 in B16 cells vs. melan-a cells. Cellular extracts (20 μg) from B16 cells and cellular extracts (40 μg) from melan-a cells (minimal amount of protein that allowed signal detection of phosphorylated ATF-2) were analyzed by western blotting using anti-ATF-2 polyclonal antibody and anti-phospho-ATF-2 polyclonal antibody, as described in Materials and Methods. The autoradiogram (top) was scanned using a Molecular Dynamics densitometer, and after correcting for the amount of total ATF-2 protein, the relative amount of phospho-ATF-2 protein in both cell lines was determined (bottom). The data shown are from a representative experiment, which was replicated three additional times with similar results.

We measured the relative expression of total ATF-2 protein in B16 and melan-a cells. Both cell lines are derived from the same genetic strain of mice (C57BL/6). B16 is a metastatic mouse melanoma cell line; whereas, melan-a is an immortalized, but non-transformed, mouse melanocyte cell line [14]. Only trace amounts of ATF-2 were detected in the cytoplasmic fraction from either cell line, with the vast majority of ATF-2 protein in the nuclear fraction. Higher levels of ATF-2 protein were detected in malignant melanoma cells, compared with non-malignant mouse melanocytes (Fig. 1A).

ATF-2 was detected as multiple bands of approximately 70 kDa in nuclear extracts from B16 and melan-a cells. The higher MW band was more prominent in the B16 nuclear extracts than that in the melan-a cell nuclear extracts. We suspected that the higher MW band was phosphorylated ATF-2. To demonstrate that ATF-2 was phosphorylated, the nuclear extracts from B16 cells were incubated with protein phosphatase-1A (PP1A) for different periods of time, and then ATF-2 protein was detected using an antibody specific for phospho-ATF-2. This experiment showed that phospho-ATF-2 was lost, even after a short incubation period (3 minutes) (Fig. 1B). In contrast, there was no loss of GAPDH immunoreactivity, indicating that the loss of phospho-ATF-2 was not due to non-specific protein degradation. Therefore, we conclude that the higher MW form of ATF-2 is highly phosphorylated in the melanoma cells.

Stimulation of ATF-2 transcriptional activity requires its phosphorylation by the MAP kinases p38 or JNK. We examined ATF-2 phosphorylation as an indicator of its activation in malignant melanoma cells, compared with non-malignant mouse melanocytes. Fig. 1C shows that, if protein content of extracts from B16 and melan-a cells is adjusted to correct for the amount of total ATF-2 expressed by these cell lines (Fig. 1A), there is a much higher amount of phospho-ATF-2 in B16 cells as compared with melan-a cells. Thus, it is likely that in addition to having more total ATF-2 protein in malignant mouse melanoma cells versus non-malignant cells, much more of the ATF-2 in melanoma cells is in the active (phosphorylated) state.

### The effect of RA on ATF-2 phosphorylation

**Figure 2:**
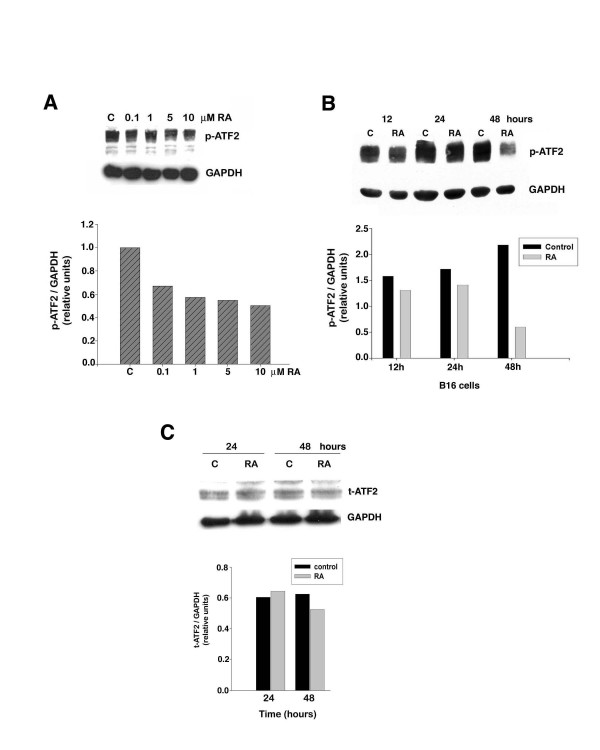
**RA inhibits ATF-2 phosphorylation in B16 mouse melanoma cells**. B16 cells were treated with either vehicle (DMSO) (C-control) or RA. At the end of the indicated incubation times, cells were harvested and protein (40 μg) from each group was analyzed by western blotting using polyclonal anti-phospho-ATF-2 or total ATF-2 antibodies as described in Materials and Methods. The relative amount of phospho- or total ATF-2 protein in each sample was determined by densitometry using GAPDH as an internal control. The data shown are from a representative experiment, which was replicated three additional times with similar results. A. Cells were treated with the indicated concentrations of RA and harvested after a 48-hour incubation. B. Cells were treated for the indicated time periods with 10 μM RA. C. The effect of different treatment times with 10 μM RA on total ATF-2 protein level.

RA increases AP-1 transcriptional activity in B16 melanoma cells. However, RA does not increase the expression of any of the Fos or Jun family members, nor does it increase the binding activity of the AP-1 complex on a TRE [12]. Because ATF-2 can form a heterodimer with c-Jun, we hypothesized that RA increases AP-1 transcriptional activity by regulating ATF-2 activation. B16 cells were treated for 48 hours with or without various concentrations of RA. At the end of the treatment period all cells were harvested and analyzed for the phospho-ATF-2 level. This experiment revealed that lowest concentration of RA tested (0.1 μM) reduced ATF-2 phosphorylation by approximately 30% (Fig. 2A). With increasing concentrations of RA the amount of phospho-ATF-2 was further reduced, falling to 50% of the control level in cells treated with the highest concentration of RA (10 μM). Treatment of B16 cells with or without 10 μM RA for different periods of time showed that RA caused a small reduction (~20%) of phospho-ATF-2 at 12 and 24 hours, but resulted in a major decrease in the phospho-ATF-2 (~75%) after 48 hours of treatment (Fig. 2B). In contrast, RA treatment caused only a small reduction in total ATF-2 protein level over 48 hours of treatment (Fig. 2C).

### Signaling pathways involved in RA-dependent inhibition in ATF-2 phosphorylation

**Figure 3:**
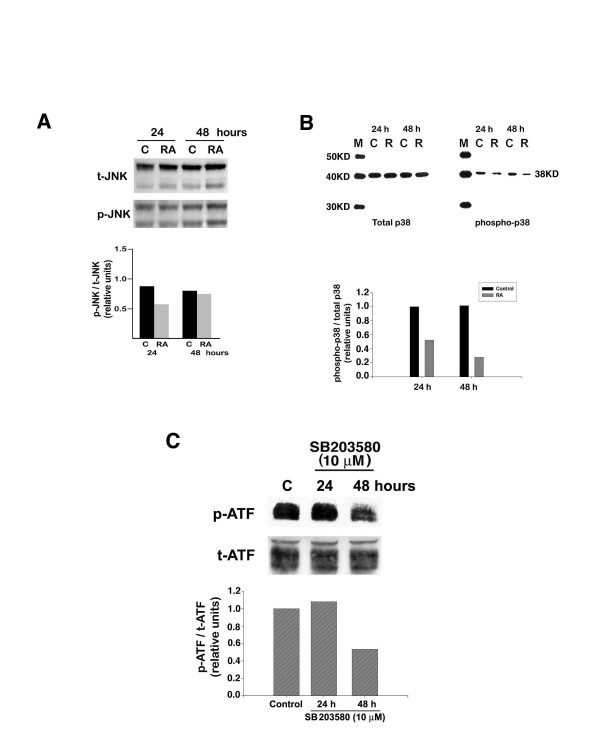
**Regulation of ATF-2 phosphorylation by p38 MAPK and inhibition by RA**. B16 cells were treated with 10 μM RA for the indicated times. Cells were then harvested, extracted, and protein from each sample examined for total and phospho-specific JNK (A) or total and phospho-specific p38 (B) by western blotting as described in Materials and Methods. The results shown are representative of three individual experiments, which gave similar results. To determine the effect of inhibition of p38 enzyme activity on ATF-2 phosphorylation, we treated cells with or without 10 μM SB203580. At the indicated times, cells were harvested, extracted, and protein from each sample was examined for phospho-specific ATF-2 using western blotting as described in Materials and Methods. The result (C) shown is representative of three individual experiments, which yielded similar results.

Both p38 and JNK MAP kinases have been implicated in stimulation of ATF-2 transcriptional activity by phosphorylation of ATF-2 protein at both Threonine-69 and -71 sites. Therefore, we investigated their potential role in RA-dependent inhibition in ATF-2 phosphorylation. We examined the state of JNK and p38 activation in control and RA-treated cells through the use of total and phospho-specific JNK and p38 antibodies. There was no consistent change in either total or phospho-specific (activated) JNK in control versus RA-treated B16 cells, at various times of RA treatment (Fig. 3A). However, RA treatment inhibited phosphorylation (activation) of p38 MAP kinase by 70% after 48 hours of treatment (Fig. 3B).

To determine whether inhibition of p38 leads to a decrease in ATF-2 phosphorylation, we treated B16 cells with or without 10 μM of the p38 MAPK selective enzyme inhibitor SB203580. This experiment showed that the SB203580 decreased the amount of phospho-ATF-2 by 50% after 48 hours of treatment (Fig. 3C). Therefore, inhibition of p38 enzyme activity parallels the effect of RA on inhibition of ATF-2 phosphorylation.

### AP-1 transcriptional activity is required for RA-dependent inhibition in ATF-2 phosphorylation

The rationale for examining ATF-2 was to discover how RA increases AP-1 activity in B16 melanoma cells. Therefore, we decided to investigate whether there is any connection between the ability of RA to inhibit ATF-2 phosphorylation and its AP-1 stimulatory activity. We have available clones of B16 cells which stably express a dominant-negative fos gene (A-fos) and have very low basal AP-1 activity, which is minimally increased by RA [13]. We investigated whether the lack of AP-1 activity might alter the ability of RA to inhibit ATF-2 phosphorylation.

**Figure 4:**
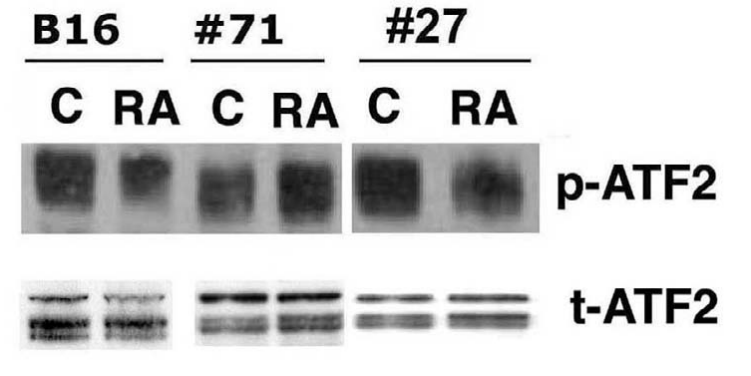
**Lack of RA-induced AP-1 activity antagonizes the ability of RA to inhibit ATF-2 phosphorylation**. Wild-type B16 cells, an A-Fos expressing clone (#71), and a non-expressing A-Fos clone (#27) were treated with or without 10 μM RA for 24 hours and 48 hours. Cells were harvested and protein (40 μg) from each group was analyzed by western blotting using total or anti-phospho-ATF-2 antibody as described in Materials and Methods. The data shown are from a representative experiment, which was replicated three additional times with similar results.

The amount of amount of total and phospho-ATF-2 protein in control and treated cells was determined by western blotting. The results indicate that, as in previous experiments, treatment of wild-type B16 cells for 48 hours with 10 μM RA inhibited ATF-2 phosphorylation by 60% (Fig. 4). However, RA treatment of a clone (#71) expressing A-fos did not result in any decrease in ATF-2 phosphorylation, while a G418-resistant clone of B16 not expressing A-Fos (#27) responded to RA treatment with the same reduction in phospho-ATF-2 levels found in wild-type B16 cells. The total amount of ATF-2 protein in wild-type cells and the clones did not substantially change subsequent to RA treatment.

### RA increases the sensitivity of B16 melanoma cells to taxol-mediated inhibition of growth

**Figure 5:**
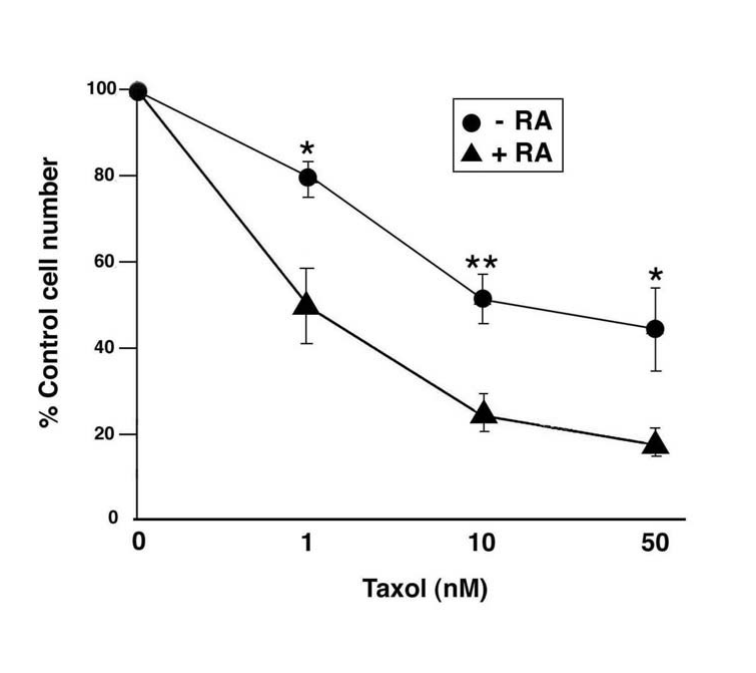
**RA increases the sensitivity of B16 cells to taxol-mediated inhibition of cell growth**. B16 cells were seeded at 2.4 × 10^4^/35 mm culture dish. Following a 24-hour attachment period, cells were treated with either vehicle (DMSO) or 10 μM RA for 48 hours. After this preincubation period, the cells were refed with growth medium containing different concentrations of taxol and incubated for 16 hours. At the end of this second incubation, the cell number in each group was determined through the use of crystal violet assay, and corrected by the initial cell number seeded. The data are presented as the mean ± SEM (error bars) of triplicate dishes and are presented as percent of control (DMSO or RA pre-treated) cell number. * p < 0.05; ** p < 0.01.

Active ATF-2 has been linked to the resistance of melanoma cells to chemo- and radiotherapeutic drugs. Therefore we hypothesized that since RA inhibited ATF-2 phosphorylation, it would increase the sensitivity of mouse melanoma cells to chemotherapeutic agents. After preliminary experiments with several cancer therapeutic drugs, we decided to focus on taxol. This chemotherapeutic compound is able to interfere with the normal progression of the cell cycle by binding to and preventing depolymerization of microtubules. It has been successfully used in the treatment of breast cancer, but its use in the treatment of melanoma has not been fully explored [15-17]. B16 cells were either treated with various concentrations of taxol for 16 hours, or pretreated with 10 μM RA for 48 hours before adding taxol. The cell number in each taxol-treated group was compared with its appropriate control (ie, pre-incubation with vehicle or 10 μM RA). We found that taxol induced a concentration-dependent inhibition in cell growth (Fig. 5). Pretreatment of cells with RA for 48 hours led to a substantial increase in sensitivity of B16 cells to taxol-induced cell growth inhibition. Without RA pretreatment,, the taxol IC _50 _was approximately 50 nM; however, when cells were pretreated with RA (10 uM) for 48 hours before adding taxol, the IC _50 _decreased to 1 nM. This action of RA correlates with its ability to inhibit the phosphorylation of ATF-2.

## Discussion

Unlike many human melanoma cell lines that are resistant to the effects of RA, B16 melanoma cells are induced by RA to undergo growth arrest in the G1 phase of the cell cycle and to increase the production of melanin, an indicator of differentiation [10]. We have previously found that treatment of B16 mouse melanoma cells with *all*-*trans*-RA induces a two to four-fold increase in AP-1 transcriptional activity, and this increase appears to be required for growth arrest and differentiation [13]. RA does not increase the expression of any of the Fos or Jun family members, nor does it increase the binding activity of the AP-1 transcription complex [12]. Therefore, we investigated the effect of RA on the expression level and phosphorylation of ATF-2, another member of the AP-1 family.

We found that ATF-2 protein was expressed at higher levels in B16 melanoma cells compared with non-malignant melan-a cells. The level of ATF-2 mRNA is also higher in some clinical samples of human tumors compared with normal tissues [18]. In melanoma, activated ATF-2 confers resistance to radiation and chemotherapeutic agents [1]. Protein analysis by Western blots demonstrated multiple bands of immunoreactive ATF-2 protein, which led us to predict that ATF-2 is a phospho-protein. Removal of the phosphate group by incubation of cell extracts with PP1 led to loss of immunoreactivity to the phospho-specific ATF-2 antibody thus confirming our hypothesis. In order for ATF-2 to form dimers and stimulate the transcription of target genes, it requires phosphorylation by the stress kinases p38 or JNK [6]. B16 cells had four-fold higher levels of phosphorylated ATF-2 compared with melan-a cells. Since there was less than a two-fold difference in the amount of total ATF-2 in these two cell lines, these data suggest that a much higher percentage of ATF-2 is in the active form in B16 compared with melan-a cells. Although high activity of ATF-2 has not been directly implicated in tumorigenesis, published data suggest that high ATF-2 activity might be important in reaching or maintaining the malignant state of melanomas [19].

A significant finding of our study is that RA decreases the phosphorylation of ATF-2 in a dose- and time-dependent manner. One other study has reported that RA alters phosphorylation of ATF-2 [20]. However, in this system, RA increased the phosphorylation and expression of ATF-2 in the early and middle phase of granulocyte differentiation, respectively. These findings are opposite to our data in melanoma and this disparity is likely due to cell type specificity.

As discussed previously, the activity of ATF-2 is regulated post-translationally by phosphorylation, particularly by the JNK/SAPK and p38 groups of mitogen-activated protein kinases [6,21-23]. Therefore, we examined the possibility that RA decreases phosphorylation of ATF-2 by interfering with the signaling pathway mediated by JNK and p38 MAP kinases. Treatment of B16 cells with RA did not alter the phosphorylation (activation) of either MEK (data not shown) or JNK. However, RA treatment resulted in a time-dependent decrease in the phosphorylation of p38, which correlated with the decrease in phosphorylation of ATF-2. There are a few other studies in which the effect of RA on p38 phosphorylation has been investigated. However, in all of these studies, RA increased p38 phosphorylation and activity [24-26]. Again, these results are opposite to our finding that RA inhibits the phosphorylation of p38 MAPK. It should be noted that in the published studies cited above none of the cells were neural crest derivatives. Our laboratory and others have found that treatment of neural crest derived cells such as neuroblastomas and melanomas with RA often has opposite effects on signaling pathways compared with treatment of non-neural crest-derived cells with RA [27-29]. To verify that inhibition of the p38 MAPK pathway might be the means by which RA decreased ATF-2 phosphorylation, we used the selective p38 MAP kinase inhibitor, SB203580. Treatment of B16 cells with this kinase inhibitor decreased ATF-2 phosphorylation. Therefore, our results suggest that the signaling pathway involved in RA-dependent inhibition of ATF-2 phosphorylation is most likely the p38 MAPK signaling pathway. In support of our findings, Ivanov and Ronai [30] reported that treatment of human melanoma cells, already expressing a peptide inhibitor of ATF-2, with a chemical inhibitor of p38 catalytic activity, resulted in apoptosis. This biological effect was not seen in cells treated with either agent alone.

We attempted to measure the effect of RA on ATF-2 transcriptional activity by transient transfection with the CRE (TGACGTCA)-luciferase reporter gene (data not shown). However, there was no significant change in luciferase activity between cells treated with and without RA (data not shown). There are two possibilities to explain this result. First, phospho-ATF-2 is expressed at high levels in B16 melanoma cells, and the ability of RA to inhibit ATF-2 phosphorylation is a slow process. Because the observation window for a transient transfection is short (24 hours), there might not have been sufficient time for RA to decrease ATF-2 phosphorylation to an extent which would affect its transcriptional activity. Second, the response elements TRE and CRE have only one nucleotide difference. Even though ATF-2 dimers prefer to bind to CRE, it might compete with the Jun:Fos dimer for binding with TRE. Therefore, the effect of RA on ATF-2 transcriptional activity could not be unambiguously tested by using the CRE-luciferase reporter gene in B16 cells.

Jun:Fos and Jun:ATF-2 represent two classes of AP-1 transcription complexes. Jun:Fos dimers bind with high affinity to the seven base pair consensus sequence TGAGTCA, while c-Jun:ATF-2 heterodimers bind with high affinity to degenerate ATF sites with the consensus motif TGACNTCA. In addition, Jun, Fos, and ATF family members can bind to DNA upon association with Maf [31,32] C/EBP [33], and NFκB [34]. The expression of several AP-1 regulated genes can be influenced by the relative amount of ATF and Jun. For example, Choi et al. [35] found that ATF-2 down-regulates hepatitis B virus promoter activity by competition for the AP-1 binding site and the formation of ATF-2:Jun heterodimers. We previously established clones of B16 which stably express dominant negative c-Fos (A-Fos [13]), and demonstrated that inhibition of AP-1 activity attenuated the ability of RA to inhibit anchorage-dependent, -independent growth and to stimulate melanin production [13]. We observed that the ability of RA to inhibit ATF-2 phosphorylation was lost in clones expressing A-fos and consequent low basal and RA-stimulated AP-1 activity. These experiments suggest that there is an intimate relationship between Fos-containing AP-1 dimers and ATF-2-containing dimers in B16 melanoma cells. The reason for the loss of RA inhibition of ATF-2 phosphorylation in clones expressing A-fos is not clear. We previously found that the ability of RA to increase RARβ and PKCα expression in A-fos expressing clones was normal, suggesting that A-fos and inhibition of AP-1 activity interferes with a down-stream, later event in the RA induction of differentiation and growth arrest in B16 melanoma cells [13]. Our results parallel the studies of Bhoumik et al. [36] who demonstrated that expression of a dominant-negative of c-Jun (TAM67) or treatment of the melanoma cells with JunD siRNA (which should result in decreased AP-1 activity) attenuated the sensitization of melanoma cells expressing the ATF-2 peptide inhibitor to apoptosis after treatment with anisomycin.

The hallmark of malignant melanoma is its resistance to chemotherapy and radiotherapy [37,38]. ATF-2 and its activating protein kinase, p38, have been found to play an important role in the resistance of melanoma to radiation and chemotherapy [1,29]. Hypophosphorylated or transcriptionally-inactive forms of ATF-2 elicit a silencing effect on TNFα expression, resulting in increased apoptosis [39]. When endogenous ATF-2 expression is inhibited by ATF-derived peptides, human melanoma cells are more sensitive to UV radiation or chemical treatment [2], and their growth and metastasis are also inhibited [40]. On the basis of these reports, we evaluated the ability of RA, which inhibits ATF-2 phosphorylation (activation), to alter the sensitivity of B16 melanoma cells to taxol-mediated growth suppression. We found that pretreatment of the B16 melanoma cells for 48 hours decreased the IC_50 _for taxol from 50 nM to 1 nM. The mechanism by which RA increases the sensitivity of B16 melanoma cells to taxol treatment is likely to involve ATF-2 activity, because inhibitors of p38 MAPK also sensitize B16 melanoma cells to taxol's growth inhibitory activity (data not shown). We attempted to construct a constitutively active AFT-2 cDNA in order to determine whether expression of this construct would block the ability of RA to sensitize B16 melanoma to taxol-induced growth inhibition. Unfortunately, using point mutations to substitute arg for thr 69 and 72 did not result in a constitutively active protein. Recently, Karmakar et al [41] reported that the combination of RA and taxol caused regression of glioblastoma T98G xenografts in nude mice. The combination caused greater apoptosis than either agent alone. Whether p38, MAPK, and ATF-2 are involved in mediating this effect is not known. However, in glioblastoma, this treatment decreased the activity of NF-kB, which is known to be activated by p38 MAPK in melanoma cells [42]. Thus an RA-mediated inhibition of p38 MAPK in the glioblastoma cells might play a role in sensitizing these cells to taxol.

Our current working model is that RA alters the balance between ATF-2:Jun containing dimers and Jun:Fos containing dimers. Phosphorylation of ATF-2 induces a conformation change that allows ATF-2 to form a homodimer with another ATF-2 or a heterodimer with Jun. Because B16 cells have high levels of ATF-2, the majority of which appears to be phosphorylated, we postulate that in untreated B16 cells most of the Jun is in a heterodimer with ATF-2. Treatment of B16 cells with RA decreases the phosphorylation of ATF-2, which results in less dimer formation with Jun. The "freed-up" Jun can then form a heterodimer with Fos, resulting in the increased AP-1 activity observed in RA-treated B16 cells. Shifting the balance from predominantly ATF-2:Jun dimers to a higher amount of Jun:Fos dimers could lead a change in target gene expression that reduces resistance to chemotherapeutic drugs and contributes to the pathway by which RA arrests proliferation and induces differentiation.

## Conclusion

We found that B16 mouse melanoma cells express 2–4 times more ATF-2 protein relative to non-malignant mouse melan-a cells. In addition, most of the ATF-2 protein in the melanoma cells is phosphorylated (active) vs. only a small amount of phosphorylated ATF-2 in non-malignant melanocytes. Retinoic acid treatment of B16 melanoma cells decreased ATF-2 phosphorylation likely due to the ability of RA to inhibit the phosphorylation (activation) of p38 MAPK. The inhibition of ATF-2 phosphorylation correlated with the ability of RA to sensitize the B16 melanoma cells to the growth inhibitory activity of the cancer chemotherapeutic agent taxol.

## Methods

### Cells and culture conditions

B16 mouse melanoma cells were grown in a humidified atmosphere of 5% CO_2_, 95% air at 37°C in Dulbecco's Modified Eagle's medium (DMEM). This medium contained 1g/L glucose and was supplemented with 10% heat-inactivated bovine calf serum (Hyclone, Logan, UT), 50 U/mL penicillin G and 50 μg/mL streptomycin sulfate.

Immortal mouse melanocytes, melan-a, were provided by Dr. Dorothy C. Bennett, St. George's Hospital Medical School, UK. Melan-a cells were grown in a humidified atmosphere of 10% CO_2_, 90% air at 37°C in RPMI 1640 culture medium. This medium contained 2 mM L-glutamine and was supplemented with 5% fetal calf serum, 200 nM TPA (tetradecanoyl phorbol acetate), 100 U/mL penicillin G and 100 μg/mL streptomycin sulfate.

*All-trans*-RA was obtained from Fluka Chemical Co (New York). All experiments involving the use of RA were conducted under yellow lights to prevent photo-oxidation of this retinoid. Fresh solutions of RA were prepared in DMSO for each experiment and then diluted to the final concentration in tissue culture media before adding to the cells.

SB203580 was obtained from Biomol Research Laboratories, Inc. (Plymouth Meeting, PA). A concentrated stock solution of SB203580 (10 mM) was prepared in DMSO and then stored at -20°C. This stock solution was diluted to the desired final concentration in tissue culture medium before adding to the cells.

Paclitaxel (taxol) was obtained from LC Labs (Wobum, MA). A concentrated stock solution of taxol (10 μM) was prepared in DMSO and then stored at -20°C in the dark. This stock solution was diluted to the desired final concentration in tissue culture medium before adding to the cells.

### Plasmid DNA constructs

CRE-Luciferase reporter plasmid was from PathDetect^® ^in vivo signal transduction pathway cis-reporting systems, Stratagene (La Jolla, CA). This vector contains the luciferase reporter gene driven by a basal promoter element (TATA box) joined to tandem repeats of the CRE (TGACGTCA) binding element.

### Transient transfection

Plasmid with or without the CRE consensus elements was transfected into early passage B16 cells via the Lipofectamine procedure (Gibco) together with a plasmid containing SV40-β-gal to correct for transfection efficiency. B16 cells were seeded (7 × 10^5 ^cells/dish) into 100-mm tissue culture plates 1 day prior to transfection. On the day of transfection, cells were refed with growth medium 4 hours prior to the procedure. Transfections included 3 μg of the plasmid vector pGL2 with or without CRE response element and 1 μg of pSV-β-galactosidase (Promega, Madison, WI). Cells were incubated with the plasmid DNAs for 5 hours before refeeding with regular growth medium. Twenty-four hours after transfection, cells were washed twice with PBS and treated with corresponding concentrations of RA for 24 hours before harvest. Cells were washed twice with PBS, harvested, and assayed for luciferase and β-galatosidase activity using the appropriate kits from Promega. Luciferase assays were evaluated in the linear range and values were normalized to β-galatosidase activity. All transfections were performed in triplicate dishes and the experiments were repeated three times.

### Crystal violet cell proliferation assay

A colorimetric assay employing crystal violet was used to determine cell growth. This assay is based on the staining of attached cells (viable cells) with crystal violet dye in each well [43]. After the treatment period, triplicate wells with various concentrations of taxol and a control group were fixed with methanol:acetic acid (3:1) for 1 hour at room temperature. The fixed cells were washed with 80% methanol and stained with crystal violet (0.5%) for 1 hour. Excess dye was removed by washing the wells with distilled water. Wells were air-dried and the dye was eluted with 500 μL of 10% acetic acid. Eluted dye (200 μL) from each well was transferred to 96-well plates and absorbance was measured at 570 nm with a kinetic microplate reader (Molecular Devices Corporation, Sunnyvale, CA). Absorbance values from the taxol-treated group were compared with values from the control group.

### Western blot assays

Cells were seeded at 2 × 10^5^/100 mm tissue culture dish. After a 24-hour attachment period, they were again fed with growth medium containing RA or DMSO. Cells were harvested after 24 hours or 48 hours of incubation depending on the experiment and prepared for western blotting.

For total ATF-2 protein analysis, B16 cells and melan-a cells were washed twice with PBS, harvested and cytoplasmic and nuclear extracts prepared using the Pierce NE-PER™ nuclear and cytoplasmic extraction reagents.

For western blot analysis of non-ATF-2 proteins, cells were washed with cold PBS and harvested in 250 μL of lysis buffer (10 mM Tris, PH 7.5, 1 mM EDTA, 1% glycerol, 1 μg/mL leupeptin, 1 μg/mL pepstatin, 50 μg/mL aprotinin, 0.5 mM PMSF). Cells were lysed on ice by three consecutive 10-sec sonications with a Tekmar^® ^sonic disruptor at power setting 60. The total lysate was stored at -80°C.

Protein concentration was determined by using the Pierce BCA protein assay kit. Protein (50 μg) was separated by SDS-PAGE with 10% separating and 5% stacking gels. The proteins were electrically transferred to a Hybond-C extra nitrocellulose membrane (Amersham, Chicago, IL). The membrane was incubated in blocking solution (Tris-buffered saline containing 0.2% Tween 20 and 10% nonfat dry milk, TBST) for 1 hour at room temperature. Blots were then incubated overnight at 4°C with a 1:1,000 dilution of polyclonal anti-JNK or polyclonal anti-phospho JNK, polyclonal anti-ERK1/2 or polyclonal anti-phospho ERK1/2, polyclonal anti-p38 or polyclonal anti-phospho p38, or polyclonal anti-phospho-ATF-2 antibodies (each at 1:1,000 dilution, Cell Signaling Technology, Beverly, MA) depending on the experiments. This solution was removed and the blot was washed several times in 1× TBST followed by a 1-hour incubation with 1:2,000 dilution of rabbit anti-mouse horseradish peroxidase-conjugated secondary antibody (Cell Signaling Technology), then washed several times in 1× TBST. Immunoreactive bands were visualized by use of the ECL kit from Amersham.

### Protein phosphatase-1 assay

PP1 is a Mn^2+^-dependent protein phosphatase with activity towards phosphoserine/threonine residues. Each 50-μL reaction contained: 25 μg nuclear protein isolated from B16 cells, 1 μL PP1 buffer, 1 mM MnCl_2_, and 2 units of PP1. Samples were incubated at 30°C for various times and the reaction was stopped by the addition of 5 μL of 10× sample buffer followed by boiling of the samples. Proteins were separated by SDS-PAGE with 10% separating and 5% stacking gels. The proteins were electrically transferred to a Hybond-C extra nitrocellulose membrane. Blots were then incubated overnight at 4°C with 1::Th1,000 dilution of polyclonal anti-phospho-ATF-2 antibody and 1:5,000 dilution of monoclonal anti-GAPDH antibody, respectively.

## Competing interests

The author(s) declare that they have no competing interests.

## Authors' contributions

YH performed most of the experiments described in the manuscript. JM repeated selected experiments to obtain publication quality figures. JM also attempted to construct a constitutively active ATF-2 plasmid. SM documented the different isoforms of p38 MAPK expressed in melan-a and B16 cells along with demonstrating that the putative constitutively active ATF-2 was not functional. RMN coordinated the design of experiments and prepared the final draft of the manuscript. All authors read and approved the final manuscript.
